# Supplementary figures and images for: Non-invasive specimen collections for Mycobacterium tuberculosis detection in free-ranging long-tailed macaques (Macaca fascicularis)
Source: PLoS One. 2023 Aug 24;18(8):e0289961. doi: 10.1371/journal.pone.0289961 (PMC10449189; doi:10.1371/journal.pone.0289961)

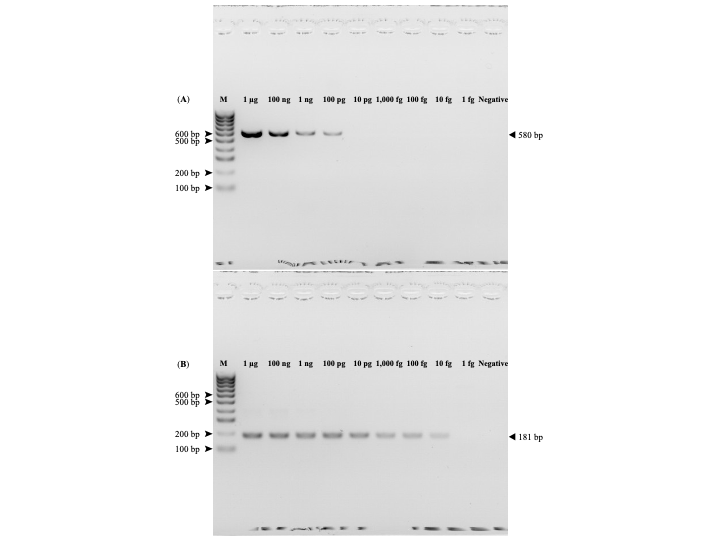

Supplement: S1 Raw images — (TIF) [file pone.0289961.s001.tif]
