# Supplementary material for: Non-invasive specimen collections for Mycobacterium tuberculosis detection in free-ranging long-tailed macaques (Macaca fascicularis)
Source: PLoS One. 2023 Aug 24;18(8):e0289961. doi: 10.1371/journal.pone.0289961 (PMC10449189; doi:10.1371/journal.pone.0289961)
Supplement: Attachment — Submitted filename: Response to Reviewers.docx [file pone.0289961.s002.docx]

**PLOS ONE**

**Manuscript No**. **# PONE-D-22-34908**

**Manuscript title**: Non-invasive specimen collections for *Mycobacterium tuberculosi*s detection in free-ranging long-tailed macaques (*Macaca fascicularis*)

**Submission as** Research article

**Authors:** Suthirote Meesawat, Nalina Aiempichitkijkarn, Saradee Warit, Mutchamon Kaewparuehaschai, Suchinda Malaivijitnond

**Response to the Editor in Chief**

Thank you very much for all of your comments to make this manuscript better. The manuscript has been thoroughly revised as indicated with yellow-highlighted.

**Reviewer: 1**

The authors presented quite a significant problem related to the collection of diagnostic material from free-living animals, especially those belonging to the group of primates. Due to the numerous restrictions related to the violation of the welfare of free-living animals and the reduction of their stress, and, on the other hand, the need to quickly identify zoonotic pathogens, the non-invasive method of sampling and its use in MTBC diagnostics seems very applicable and I certainly recommend the article for publication. However, a few clarifications still need to be made, even though the manuscript has already undergone initial revisions:
It is not clear why the material was taken again after 5 days using the invasive method. Isn't this too short a time for animals to fully recover after pharmacological treatment? Please justify such a short time as well as repeating the collection with a non-invasive method on the 2nd, 3rd and 4th day.

**Ans**: For clarification, these animals did not receive any pharmacological treatment, they were only subjected to biological specimen collection for MTBCS detection. The invasive specimen collection was done twice on Day-1 and Day-2, and non-invasive specimen collection was done thrice on Day-2, 3 and 4. These multiple collections in a single animal during a short period of time were done because we wanted to confirm that there were no technical errors of specimen collection on MTBCS negative results. Since all repetitive collected specimens (both invasive and non-invasive methods) showed the same results throughout the 2 and 3-day periods, this indicates that the negative results were not the specimen collection error, but the animals were no MTBCS.

Note, this explanation was also added in the Discussion (Page 16 and 17) of the revised manuscript.

I also believe that the method of collecting material from free-living animals populations is described too generally. Animal populations were studied, but in what space? It is hard to imagine that, as in the case of animals kept in cages, faeces were collected immediately after defecation, or a rope was found with the certainty that it had been chewed by the animal - please specify this part of the experiment.

**Ans**: The habitat type of each population and the GPS of each location were added (Page 10). The procedures of baiting the 20% sucrose ropes to animals and collecting of the discarded chewed rope were added into the manuscript. The procedure of following monkey before collection of the freshly defecated excretions was also added (Page 10 and 11).

Were the results of the research supported by microbiological culture or were they based only on the results of molecular tests? If not, please explain in the text why.

**Ans**: The results were based only on the molecular test, and we did not perform the culture for the following reasons.

i)For fecal specimen: Although the fecal swab was done in the inner part of the feces and had low possibility to be contaminated with ground pathogens, it can not be used for culture because it was highly contaminated with gut microflora.

ii)For oral specimen: The amount of MBTCS is too low for culture and it was contaminated with other pathogens on the ground that could interfere with the growth of the MTBC.

We did not add this explanation in the manuscript because we thought that our writing in the introduction and methods was clear enough to convey our intention that we aimed to use only IS*6110*-nested PCR method for MTBCS detection. We are also afraid that adding the explanation why we did not confirm the nested-PCR results by microbiological culture will cause confusion to the readers.
